# Mainstreaming Mistrust: The Shift in US Vaccine Policy Under the Trump Administration

**DOI:** 10.3389/ijph.2026.1609574

**Published:** 2026-03-05

**Authors:** Sanjin Musa, Amy Estlund, Megan A. Berman

**Affiliations:** 1 Institute for Public Health of the Federation of Bosnia and Herzegovina, Sarajevo, Bosnia and Herzegovina; 2 Sarajevo School of Science and Technology, Sarajevo, Bosnia and Herzegovina; 3 Fulbright US Scholar to Bosnia and Herzegovina, Lindenwood University, St. Charles, MO, United States; 4 Department of Internal Medicine, Sealy Institute for Vaccine Sciences, University of Texas Medical Branch, Galveston, TX, United States

**Keywords:** global burden, hesitancy, immunization agenda 2030, policy, vaccine

Poliomyelitis emerged as a major public health threat in the mid-20th century, when near-annual epidemics caused substantial seasonal outbreaks and terror across Western Europe and North America. During this period, images of children in leg braces and crowded hospital wards lined with “iron lungs” became powerful symbols of public fear and collective vulnerability. Swimming pools and movie theaters were often closed during the summer months as parents sought to protect their children from an invisible threat that struck without warning. Against this backdrop, the March of Dimes mobilized widespread community engagement and support, galvanized in part by President Franklin D. Roosevelt, himself a prominent public face of polio. The Foundation harnessed American generosity and a shared sense of purpose to raise funds for research, education, and ultimately, vaccine development. The development of a polio vaccine became highly competitive, with multiple researchers and institutions working to produce a safe and effective immunization. Ultimately, two vaccines were licensed and widely adopted: the inactivated poliovirus vaccine (IPV), developed by Jonas Salk at the University of Pittsburgh in 1955, and the oral poliovirus vaccine (OPV), developed by Albert Sabin at the University of Cincinnati in 1961 [1].

The triumph of these vaccines transformed public fear into collective relief, marking one of the greatest achievements in modern medicine. Given the historic role of vaccines in safeguarding humanity from devastating infectious diseases, the world is watching with concern as the United States undergoes a significant reversal in its vaccine policies driven by the current administration. This shift carries potentially far-reaching consequences that threaten to undermine both US and global immunization efforts. Although anti-vaccine activism has historically remained on the margins of public discourse, this landscape is shifting as Robert F. Kennedy, Jr (RFK Jr) has assumed leadership of the Department of Health and Human Services (HHS).

Controlling infectious diseases has always required a collective approach because infections extend beyond the individual and carry the potential for widespread transmission. The world united to combat polio, rallying around the proven and effective solution of vaccination through a coordinated global effort. Several governmental and non-governmental organizations worked toward universal immunization. Since its launch in 1988, the Global Polio Eradication Initiative (GPEI) has made remarkable progress, reducing the number of children paralyzed by polio by 99.9% [2]. The joint efforts of Rotary International, WHO (World Health Organization), GPEI, the Gates Foundation, CDC (US Centers for Disease Control and Prevention), United Nations International Children’s Emergency Fund (UNICEF), and Gavi, the Vaccine Alliance showed the power of coordinated action and unified goals. Philanthropic contributions supported research and treatment infrastructure, while mass communication campaigns and media coverage effectively energized public support and engagement.

Recent policies under the Trump administration, more specifically within HHS under RFK Jr., have generated public confusion and concern. These shifts include the immediate replacement of all 17 members of the Advisory Committee on Immunization Practices (ACIP), the departure of several senior CDC officials, public questioning of vaccine safety, alterations to established childhood immunization schedules, cancellation of funding for promising mRNA vaccine platforms, and stopping financial support to the WHO and Gavi, the Vaccine Alliance. RFK Jr. received US Senate confirmation for his role at the HHS despite a well-established reputation for anti-vaccine activism, and his initial actions in office have only reinforced these concerns.

The Immunization Agenda 2030 (IA 2030) is a global strategy and advocacy tool designed to guide immunization efforts from 2020 to 2030 [3]. It was introduced in April 2020 amid the severe disruptions to routine immunization services caused by the COVID-19 pandemic. The rapid development and deployment of COVID-19 vaccines was unprecedented, preventing an estimated 14.4 million deaths and reducing global mortality by 79% in the first year of vaccination [4]. However, the pandemic also led to a nearly 40% increase in the number of zero-dose children (not receiving even a single vaccine), rising from 13.3 million in 2019 to 18.2 million in 2021 [5]. These developments underscore both the progress and setbacks of recent years, emphasizing the need for IA 2030 to strengthen global immunization efforts.

The overarching strategic priority of IA 2030 is to ensure that immunization programs are an integral part of primary healthcare to achieve universal coverage. The COVID-19 pandemic highlighted the importance of primary healthcare and revealed both strengths and vulnerabilities in health systems worldwide. Studies from low- and middle-income countries across all six WHO regions have documented the profound impact of the pandemic on countries’ health system functioning [6]. Common challenges include gaps in governance and leadership, imbalances in human resources, mistrust between communities and healthcare providers, persistent geographic and socioeconomic inequities, and insufficient health financing to support system resilience. As the vaccine landscape and public expectations continue to evolve, healthcare providers require additional support. For decades, ACIP and CDC have served as vital and credible sources of guidance for institutions and agencies involved in public health globally.

In the US, political interference in vaccine policy and shifts in scientific leadership have raised concerns among the medical and public health communities, threatening evidence-based decisions and global cooperation when strong leadership is essential [7, 8]. Undermining the authority and expertise of national public health institutions not only fails to foster informed public discourse but also increases the risk of amplifying anti-vaccine activism and facilitating the spread of misinformation [8].

Vaccine hesitancy, which has existed since the earliest days of vaccination, continues to evolve according to context, geography, and vaccine type. The WHO identifies vaccine hesitancy as a major threat to global health. Vaccine hesitancy, defined as delayed acceptance or refusal of vaccines despite their availability [9], is influenced by factors such as complacency, convenience, and confidence. Although extensive safety and efficacy data are available in peer-reviewed literature and reports from international health authorities and independent professional groups, the central challenge often lies in the willingness to accept scientific evidence. Confidence depends on trust in vaccine safety and effectiveness, in the health system that delivers vaccines, and in the motivations of policymakers who recommend them [9].

The full global consequences of these policy shifts are not yet known, but they are likely to undermine public trust by fueling vaccine misinformation, potentially decreasing vaccine coverage, and weakening international health collaborations—ultimately putting populations worldwide at risk. In the face of these challenges, the response of health professionals and the scientific community will be critical. One example is the independent analysis of the recent ACIP meeting, published in *Vaccine* in November 2025 [10]. We believe it is essential not only to address public concerns and counter misinformation, but also to protect the integrity of key public health institutions and preserve the trust that is fundamental to successful immunization programs.
